# Supplementary material for: Fabrication of a Novel CNT-COO−/Ag3PO4@AgIO4Composite with Enhanced Photocatalytic Activity under Natural Sunlight
Source: Molecules. 2023 Feb 7;28(4):1586. doi: 10.3390/molecules28041586 (PMC9967086; doi:10.3390/molecules28041586)
Supplement: Supplementary file 1 [file molecules-28-01586-s001.zip › molecules-2156489-supplementary.pdf]

Supporting Information for

Fabrication of a novel CNT-COO^-/Ag_3PO_4@$AgIO_4$Composite with Enhanced Photocatalytic Activityunder Natural Sunlight

1. Characterization

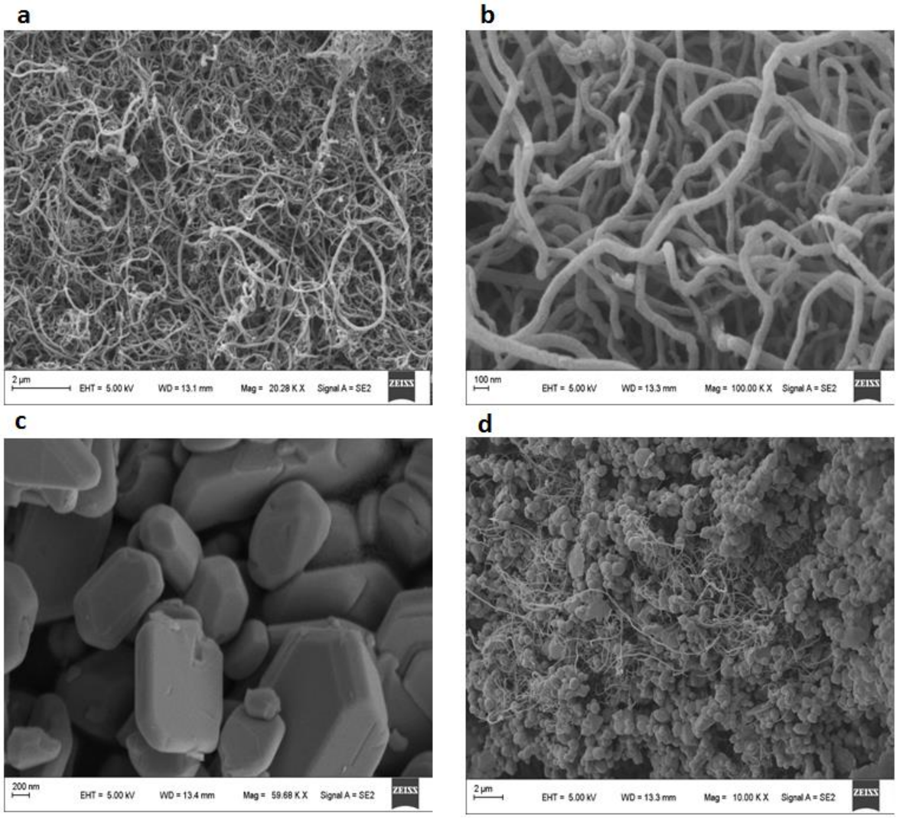

Figure S1- SEM image of (a) nonpurified CNT, (b) purified CNT, (c) $AgIO_4$, (d) CNT/Ag_3PO_4@$AgIO_4$.

2. Photocatalytic Degradation Results and Analysis.

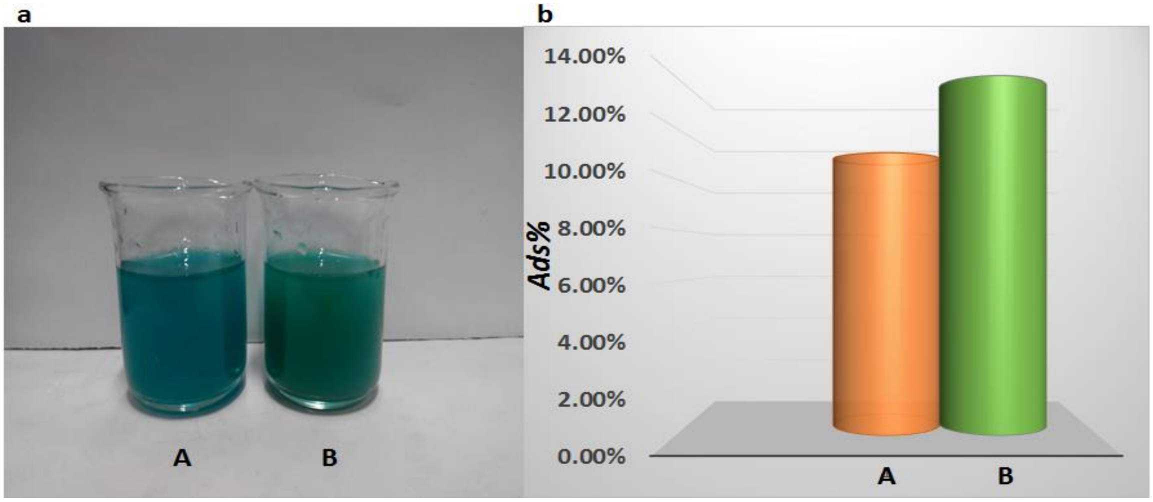

Figure S2- (a) Image of the dispersion of (A) CNT/Ag_3PO_4@$AgIO_4$ and (B) CNT-COO/Ag_3PO_4@$AgIO_4$, in MB aqueous solution. (b) The adsorption of MB over: (A) CNT/Ag_3PO_4@$AgIO_4$ and (B) CNT-COO/Ag_3PO_4@$AgIO_4$ composites.

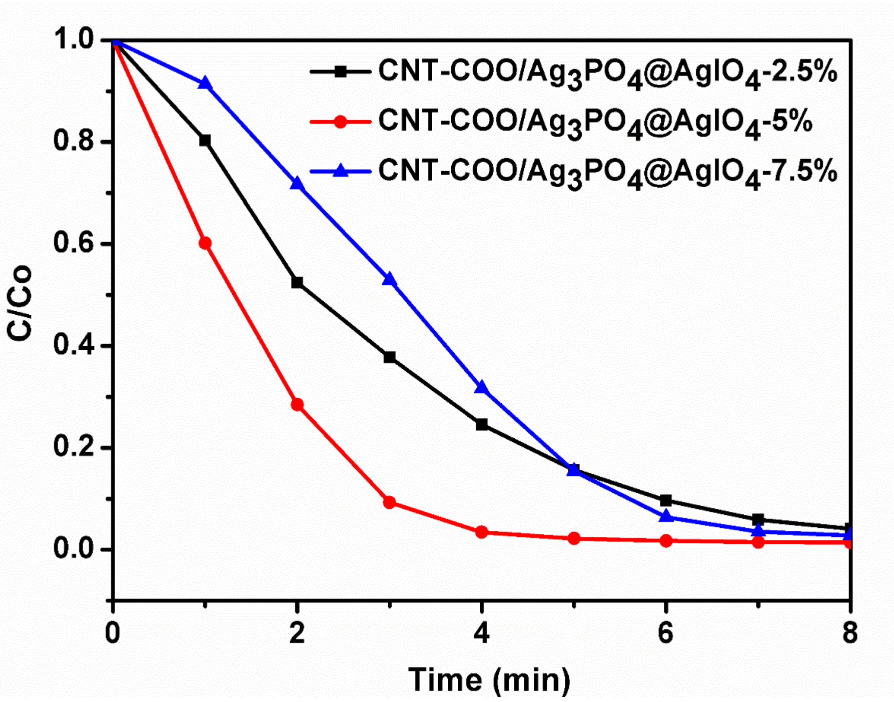

Figure S3- The photodegradation of MB over CNT-COO/Ag_3PO_4@$AgIO_4$with different CNT-COO contents.

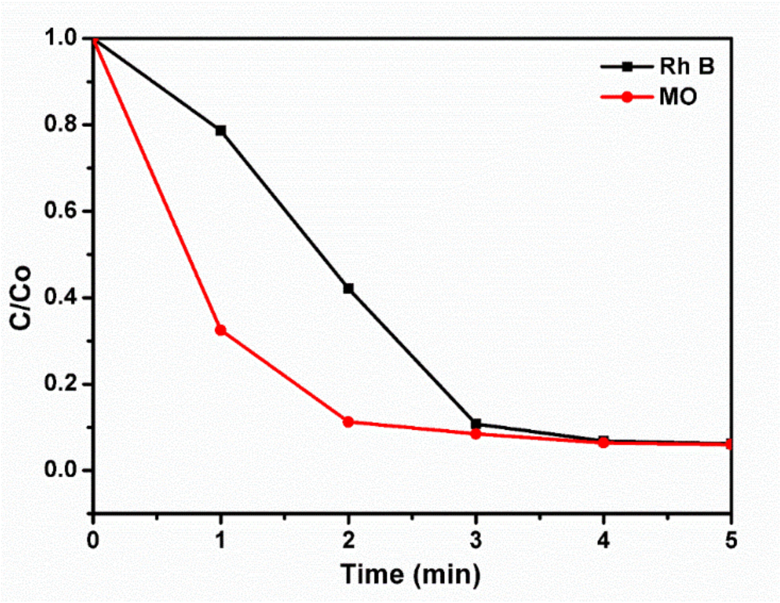

Figure S4- The photodegradation of different organic dyes over CNT-COO/Ag_3PO_4@$AgIO_4$-5%, under sunlight irradiation.

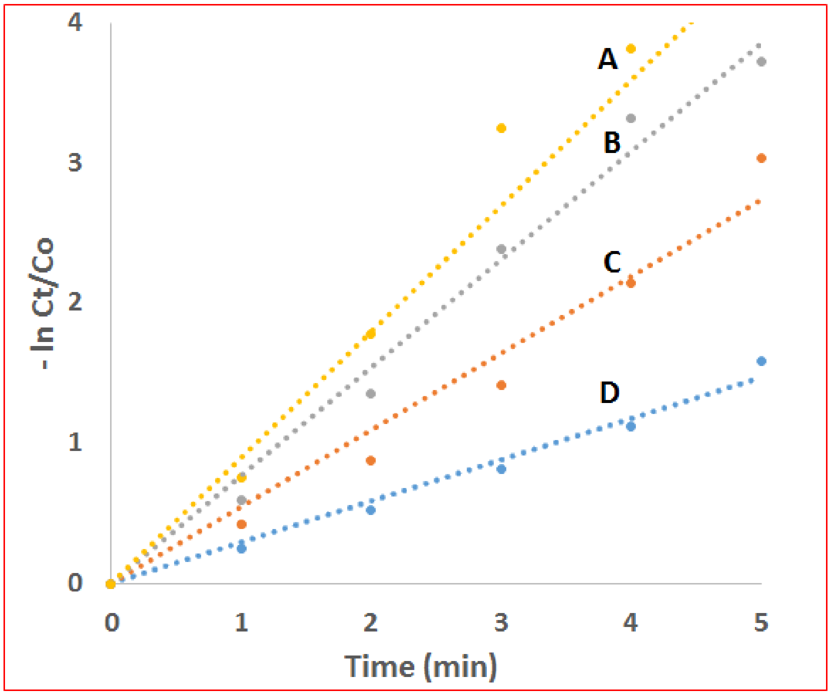

Figure S5- Regression curves of – ln(Ct/Co) versus irradiation time for CNT-COO/Ag_3PO_4@$AgIO_4$-5% under different light intensities (A) 100%, (B) 75%, (C) 50%, (D) 25%.
